# Integrated multi-omics analysis reveals the functional and prognostic significance of lactylation-related gene PRDX1 in breast cancer

**DOI:** 10.3389/fmolb.2025.1580622

**Published:** 2025-04-04

**Authors:** Qinqing Wu, Heng Cao, Jiangdong Jin, Dongxu Ma, Yixiao Niu, Yanping Yu, Xiang Wang, Yiqin Xia

**Affiliations:** ^1^ Department of Preventive Medicine, Shantou University Medical College, Shantou, China; ^2^ School of Public Health, Shantou University, Shantou, China; ^3^ Department of Breast Surgical Oncology, National Cancer Center/National Clinical Research Center for Cancer/Cancer Hospital, Chinese Academy of Medical Sciences and Peking Union Medical College, Beijing, China; ^4^ Department of Breast Surgery, The First Affiliated Hospital of Nanjing Medical University, Nanjing, China

**Keywords:** breast cancer, PRDX1, lactylation, spatial transcriptomics, prognostic model

## Abstract

**Background:**

Breast cancer (BRCA) is a significant threat to women’s health worldwide, and its progression is closely associated with the tumor microenvironment and gene regulation. Lactylation modification, as a key epigenetic mechanism in cancer biology, has not yet been fully elucidated in the context of BRCA. This study examines the regulatory mechanisms of lactylation-related genes (LRGs), specifically PRDX1, and their prognostic significance in BRCA.

**Methods:**

We integrated data from multiple databases, including Genome-Wide Association Study (GWAS) summary statistics, single-cell RNA sequencing, spatial transcriptomics, and bulk RNA sequencing data from The Cancer Genome Atlas (TCGA) and Gene Expression Omnibus (GEO) databases. Using Summary-based Mendelian Randomization (SMR) analysis, we identified LRGs associated with BRCA and comprehensively analysed the expression patterns of PRDX1, cell-cell communication networks, and spatial heterogeneity. Furthermore, we constructed and validated a prognostic model based on the gene expression profile of PRDX1-positive monocytes, evaluating it through Cox regression and LASSO regression analyses.

**Results:**

PRDX1 was identified as a key LRG significantly associated with BRCA risk (p_SMR = 0.0026). Single-cell RNA sequencing analysis revealed a significant upregulation of PRDX1 expression in monocytes, with enhanced cell-cell communication between PRDX1-positive monocytes and fibroblasts. Spatial transcriptomics analysis uncovered heterogeneous expression of PRDX1 in the tumor nest regions, highlighting the spatial interaction between PRDX1-positive monocytes and fibroblasts. The prognostic model constructed based on the gene expression profile of PRDX1-positive monocytes demonstrated high accuracy in predicting patient survival in both the training and validation cohorts. High-risk patients exhibited immune-suppressive microenvironment characteristics, including reduced immune cell infiltration and upregulation of immune checkpoint gene expression.

**Conclusion:**

This study reveals the key role of PRDX1 in BRCA progression, mainly through the regulation of the tumor microenvironment and immune escape mechanisms. The survival prediction model based on PRDX1 shows robust prognostic potential, and future research should focus on integrating PRDX1 with other biomarkers to enhance the precision of personalised medicine.

## 1 Introduction

Breast cancer (BRCA) is the most common malignancy among women worldwide, with both incidence and mortality rates rising annually. According to the World Health Organization’s 2022 cancer statistics, BRCA ranks second in incidence among all cancer types, with 2,308,897 new cases reported. It is the leading cause of cancer-related deaths in women, with 665,684 new deaths attributed to BRCA (Bray et al., 2024; Giaquinto et al., 2024), significantly impacting women’s health and quality of life. Despite the increasing standardisation and personalisation of treatments, including the widespread use of early screening and the combination of surgery, radiotherapy, chemotherapy, targeted therapies, and immunotherapy, which have improved overall treatment outcomes (Gradishar et al., 2024; His et al., 2024; Wheeler et al., 2024), the complexity and heterogeneity of BRCA make its prognosis still concerning. In recent years, the tumor microenvironment (TME) of BRCA has become a research hotspot, with its impact on tumor initiation, progression, and therapeutic response being increasingly elucidated. The TME of BRCA consists of tumor cells, immune cells (such as tumor-associated macrophages, CD8^+^ T cells, and regulatory T cells), fibroblasts, and various cytokines and metabolic products. Studies have shown that BRCA can achieve immune evasion through multiple mechanisms, including the upregulation of immune checkpoint molecules (such as PD-L1 and CTLA-4), alteration of antigen presentation capabilities, and reprogramming of tumor-associated macrophage (TAM) phenotypes (Zhang et al., 2024). Therefore, investigating the dynamic changes in the BRCA immune microenvironment and understanding how metabolic regulation influences immune evasion mechanisms are crucial for optimising immunotherapy strategies.

Lactylation modification is a novel post-translational modification (PTM) that has garnered significant attention in recent years (Zhang et al., 2019). The discovery of this modification challenges the traditional understanding of lactate’s function, which was once considered merely a byproduct of glycolysis. Increasing evidence suggests that lactate, as a metabolic feedback regulator and a unique signalling molecule, participates in various biological processes, including regulating energy metabolism, immune responses, memory formation, wound healing, and tumor development (Chen et al., 2021; Li et al., 2022; Yu et al., 2024). It is critical in cellular metabolism and gene expression regulation (Izzo and Wellen, 2019). Lactate-mediated lactylation can be categorised into histone lactylation and non-histone lactylation. Histone lactylation emphasises the cross-regulation between metabolism and epigenetics. In contrast, non-histone lactylation is more directly involved in regulating metabolic pathways and signalling, which are closely associated with tumor initiation and progression (Li et al., 2024; Chen et al., 2025). In ocular melanoma, histone lactylation accelerates tumor progression by promoting the expression of the m6A reader protein YTHDF2 (Yu et al., 2021). Gao et al. found that non-histone lactylation is prevalent in hepatitis B virus-related hepatocellular carcinoma (HCC) and may promote HCC progression (Yang et al., 2023). Colorectal cancer (CRC) also exhibits a close relationship between lactylation modification and tumor metabolic reprogramming. Under hypoxic conditions, lactate accumulation promotes the lactylation of key metabolic enzymes and signalling pathway proteins, thereby enhancing tumor cell survival ability (Xie et al., 2024). In BRCA, tumor cells and cancer-associated fibroblasts (CAFs) enhance glycolysis, leading to a significant increase in extracellular lactate concentration (up to 10–40 mM) (Zhang Y. et al., 2023). The lactate produced by tumors can trigger lactylation of histone H3K18 at the c-Myc promoter region, remodelling the epigenetic regulatory network to promote BRCA growth and proliferation (Pandkar et al., 2023). Lactylation modification offers a new perspective for cancer treatment strategies and has been proposed as a novel therapeutic target in oncology (Dai et al., 2022). However, although the regulatory role of lactylation in BRCA has been preliminarily explored, the diagnostic value of its key effector genes, underlying molecular mechanisms, and interactions with the tumor microenvironment remain insufficiently elucidated and require further investigation.

Summary-based Mendelian Randomization (SMR) is a method of Mendelian randomisation analysis based on summary data used to assess whether there is a causal relationship between gene expression and complex diseases. This approach combines genetic instrumental variables for gene expression with Genome-Wide Association Study (GWAS) data for diseases, using statistical analysis to infer whether gene expression has a causal impact on the disease (Evans and Davey Smith, 2015). Single-cell sequencing is a high-throughput technology that allows the analysis of genomic, transcriptomic, or epigenomic information at the single-cell level. Unlike traditional bulk sequencing, it reveals cellular heterogeneity, identifies rare cell subpopulations, and analyzes dynamic changes in cell development trajectories and functional states (Haque et al., 2017). Spatial transcriptomics is a high-throughput sequencing technology that combines gene expression with spatial location information. It can retain the tissue’s original spatial structure while analysing gene expression profiles of cells in different regions, revealing the relationship between gene activity and the cellular microenvironment (Marx, 2021).

In the process of screening and validating biomarkers for BRCA, confounding factors such as sample selection bias, intra-/inter-tumor heterogeneity, dynamic fluctuations in the lactate microenvironment, and platform-specific biases in epigenetic analyses can significantly impact the accuracy of research conclusions and their clinical translation potential. Therefore, identifying and analysing lactylation-related genes (LRGs) through multi-omics approaches are critical for optimising molecular subtyping and developing targeted treatment strategies for BRCA. This study combined Mendelian randomisation, single-cell sequencing, and spatial transcriptomics to identify and analyse the BRCA-associated LRG PRDX1, unveiling its regulatory mechanisms and prognostic value. This provides a theoretical foundation and practical guidance for the early diagnosis and personalised treatment of BRCA.

## 2 Materials and methods

### 2.1 Data collection and preprocessing

The GWAS summary data for BRCA were obtained from the Integrative Epidemiology Unit (IEU) Open GWAS Database (https://gwas.ac.uk/), with a study population of European ancestry and GWAS ID “ieu-a-1126” (Zhang and Tang, 2025). Based on previous studies on lactylation modification, we compiled and selected 327 genes associated with lactylation modification (Liu H. et al., 2025; Xu et al., 2025). The single-cell transcriptomic data used in this study were sourced from the Gene Expression Omnibus (GEO) database, specifically the GSE161529 dataset (https://www.ncbi.nlm.nih.gov/geo/), which comprises 16 samples. This dataset includes a single-cell transcriptomic map for nearly 120,000 cells. The spatial transcriptomic sample data were obtained from the “Human Breast Cancer: Visium Fresh Frozen, Whole Transcriptome” dataset provided by 10x Genomics (https://www.10xgenomics.com/) (Bock et al., 2025). The TCGA-BRCA transcriptome data (TPM) and clinical information were retrieved from the UCSC Xena platform (http://xena.ucsc.edu/), including 1,118 BRCA and 113 standard breast tissue samples. The GSE42568 dataset was downloaded using the GEOquery package in R, containing gene expression matrices and clinical information for 104 BRCA patients and 17 standard breast tissue samples. We merged the downloaded expression matrices by column and applied normalisation and log2 transformation to improve the data distribution characteristics (Zhao et al., 2024). Additionally, we used annotation files to convert probe IDs to gene symbols, averaging values for probes corresponding to the same gene to ensure data consistency.

### 2.2 Colocalization exploration of BRCA pathogenic genes and LRGs

We utilised SMR software version 1.3.1 for Mendelian randomisation analysis to explore causal relationships between gene expression and disease (Duan et al., 2024). The study was based on the quality-controlled 1000 Genomes Project European genetic data, with a minimum allele frequency (MAF) of 1% and a differential allele frequency of 99%. To precisely select genes, we retained those with p-values less than 0.05 to ensure statistical significance. Next, we further selected all genes annotated as “protein_coding.” To explore the role of lactylation modification in BRCA, we performed an intersection analysis between these protein-coding genes and a known set of LRGs, identifying LRGs associated with BRCA (Yang et al., 2025).

### 2.3 Cell type and communication feature analysis of BRCA 10x gene expression data

We performed a multi-step analysis of the BRCA 10x gene expression data to explore cell type and communication features. First, we performed quality control using the subset function to filter low-quality cells. We retained cells with gene counts between 200 and 2,500 to exclude empty droplets and multiplets. Cells with over 20% mitochondrial gene expression were removed to eliminate dying or stressed cells, and those with more than 5% haemoglobin gene expression were excluded to avoid red blood cell contamination. Next, we normalised the data using NormalizeData to ensure comparability of gene expression across different cells. We then selected 3,000 highly variable genes using the variance-stabilizing transformation (VST) method, implemented in the FindVariableFeatures function of the Seurat package, aiming to capture sufficient biological variation for cell population analysis while avoiding over-complexity. Principal component analysis (PCA) was conducted, and the optimal number of principal components was determined using the ElbowPlot method to extract the main components, thereby providing a low-dimensional representation for subsequent cell clustering analysis (Zhang P. et al., 2023). We performed cell clustering using FindNeighbors and FindClusters, identifying cell populations with similar gene expression profiles, which laid the foundation for cell type annotation and communication analysis (Yao et al., 2025). Cell types were annotated using the SingleR method, based on comparisons with a reference dataset (Human Primary Cell Atlas) to infer each cell type. To analyse cell-cell communication, we used the CellChat package to construct ligand-receptor interaction networks, assess communication probabilities, and identify significant communication pathways (Bai et al., 2025). Finally, to study the dynamic changes in monocytes at different states, we conducted pseudotime analysis using Principal Component Analysis (PCA) and Uniform Manifold Approximation and Projection (UMAP) for dimensionality reduction. We applied the Harmony package to correct for batch effects, thereby ensuring the reliability of the results.

### 2.4 Cell population analysis and spatial interaction pattern exploration of BRCA spatial transcriptomic data

First, we used the Seurat package to load and preprocess the BRCA spatial transcriptomic data, extracting tissue coordinates and performing quality control (Bai et al., 2025). We filtered out data points outside the tissue and removed low-quality spots, such as those with mitochondrial or ribosomal gene expression and fewer than 10 genes. The data were then normalised and subjected to dimensionality reduction (PCA and UMAP) to prepare for further cell population analysis and visualisation. Next, we performed a deconvolution analysis of the BRCA spatial data using the RCTD method, integrating spatial data with single-cell RNA reference data to infer the cellular composition at each spatial location (Zhang W. et al., 2025). The results were stored in a deconvolution matrix and used for the quantitative analysis of cell types.

Using this foundation, we employed the mistyR package to infer the spatial interactions between cells and analysed the spatial interaction patterns between different cell types. We systematically revealed the spatial relationships between cell types by combining spatial transcriptomic data and deconvolution analysis. Initially, we performed a kNN analysis to calculate the six nearest neighbours for each spot and selected spots that were positive for PRDX1 in monocytic cells. Based on these spots, we constructed a spatial network. We calculated the degree of each spot, generating a homotypic cell interaction network that visually demonstrated the spatial distribution and interrelations of PRDX1-positive monocytic cells. Furthermore, we generated a heterotypic cell interaction network for PRDX1-positive and negative monocytic cells interacting with fibroblasts. These networks display the spatial distribution of various cell types and reveal their intricate interrelationships, offering crucial insights into the spatial interactions between cells. Finally, we performed neighbourhood enrichment analysis, calculated the enrichment of cell types among neighbours, and visualised and standardised the results (Salié et al., 2025).

### 2.5 Construction and validation of a survival prediction model for BRCA

In the filtered single-cell data, we used the FindMarkers function to identify differentially expressed genes (logfc = 0.6) associated with PRDX1-positive monocytic cells. We selected the TCGA dataset (n = 1,231) as the training set due to its large sample size and comprehensive molecular characteristics, while the GEO dataset (n = 121) was used as an external validation set. To eliminate batch effects, we applied the SVM method for correction. Subsequently, univariate Cox regression analysis was performed to evaluate the association between genes and survival prognosis. To further identify key genes, we employed Lasso regression, using L1 regularisation to reduce multicollinearity and enhance model stability. 10-fold cross-validation was used to determine the optimal lambda value, minimising error and improving model generalizability (Gómez et al., 2025). A multivariate Cox regression model was further constructed to identify the best feature genes. The Cox regression model derived from the training set was applied to survival prediction in the validation set. Finally, Kaplan-Meier survival curves were generated using the survival package, and time-dependent receiver operating characteristic (tROC) curves were plotted using the timeROC package to assess the model’s accuracy in predicting patient survival.

### 2.6 Cox regression analysis of survival factors in BRCA patients

We performed univariate and multivariate Cox regression analyses to evaluate the influence of various clinical factors on the survival of BRCA patients. These analyses encompassed age, T stage, N stage, M stage, and risk score (Pei et al., 2023). The results of the regression analyses were visually presented through forest plots, which depicted the hazard ratios (HRs) and corresponding confidence intervals for each variable. Additionally, Kaplan-Meier survival curves were used to compare the survival outcomes among different risk groups. We also conducted subgroup analyses to examine the impact of specific clinical factors on survival. The significance of all findings was determined using p-values and confidence intervals.

### 2.7 Comprehensive analysis of immune microenvironment and biological pathways in BRCA

To investigate the immune landscape in BRCA, we utilised the MCPcounter method to quantify the relative abundance of various immune cell types within the tumor microenvironment. Boxplots and density plots illustrated the disparities in immune cell infiltration between high-risk and low-risk groups. Additionally, we employed the Estimate method to compute immune scores and tumor purity, which facilitated a deeper understanding of how the immune microenvironment influences patient survival outcomes (Ye et al., 2025). Furthermore, Single-sample Gene Set Enrichment Analysis (ssGSEA) was conducted to assess the activation levels of diverse biological pathways in BRCA samples, highlighting the significance of immune-related pathways in survival prognosis. Lastly, we identified key checkpoint genes and utilised boxplots and density plots to elucidate the expression differences of these genes between high-risk and low-risk cohorts.

### 2.8 Cell transfection

The Cell Resource Center of Shanghai Life Sciences Institute provided the MDA-MB-231 and HCC1806 cell lines. The primers used in this study were designed and synthesised by Qingdao Biotechnology, located in Beijing, China. Additionally, the PRDX1-targeting siRNA and its corresponding negative control (Si-NC) were provided by RiboBio, based in Guangzhou, China. Detailed sequences of the primers and siRNA are provided in Supplementary Table S1.

### 2.9 Healing

For the wound-healing assay, transfected cells were cultured in 6-well plates until they nearly reached 95% confluence. Scratches were created in each well using a sterile 200 μL plastic pipette tip, and the wells were then washed twice with PBS to remove floating cells and debris. Serum-free medium was subsequently added. Images of the scratches were taken at 0 h and after 48 h, and the width of the scratches was measured using ImageJ software.

### 2.10 Transwell

In the Transwell assay, 4 × 10^4^ transfected cells were placed in the upper chamber of a 24-well plate and incubated for 48 h. The plate’s bottom was either treated with Matrigel solution (BD Biosciences, USA) or left untreated to assess the cells’ potential for invasion and migration. Once the medium was removed, the cells were washed twice with PBS, fixed at room temperature for 30 min in 4% paraformaldehyde, and then washed again with PBS. Next, 0.1% crystal violet (Solarbio, China) was added for a one-hour staining period. Afterwards, the cells were rinsed until the background was transparent, and any cells on the top surface of the pores were carefully removed using cotton swabs. Finally, ImageJ software is used to count.

### 2.11 Statistical analysis

In this study, data were standardised using log transformation and batch correction. All analyses and visualisations were conducted with R software (version 4.3.3). A predictive model was developed through a series of statistical methods, including univariate, Lasso, and multivariate Cox regression analyses. For statistical comparisons, t-tests were used to assess differences between two groups, while one-way ANOVA was applied to evaluate differences among multiple groups. The Wilcoxon test was explicitly used to compare high- and low-risk groups. Statistical significance was defined as a p-value of less than 0.05, with significance levels denoted as follows: * (p < 0.05), ** (p < 0.01), and *** (p < 0.001).

## 3 Results

### 3.1 Interaction and relationship analysis between BRCA and LRGs

**FIGURE 1:**
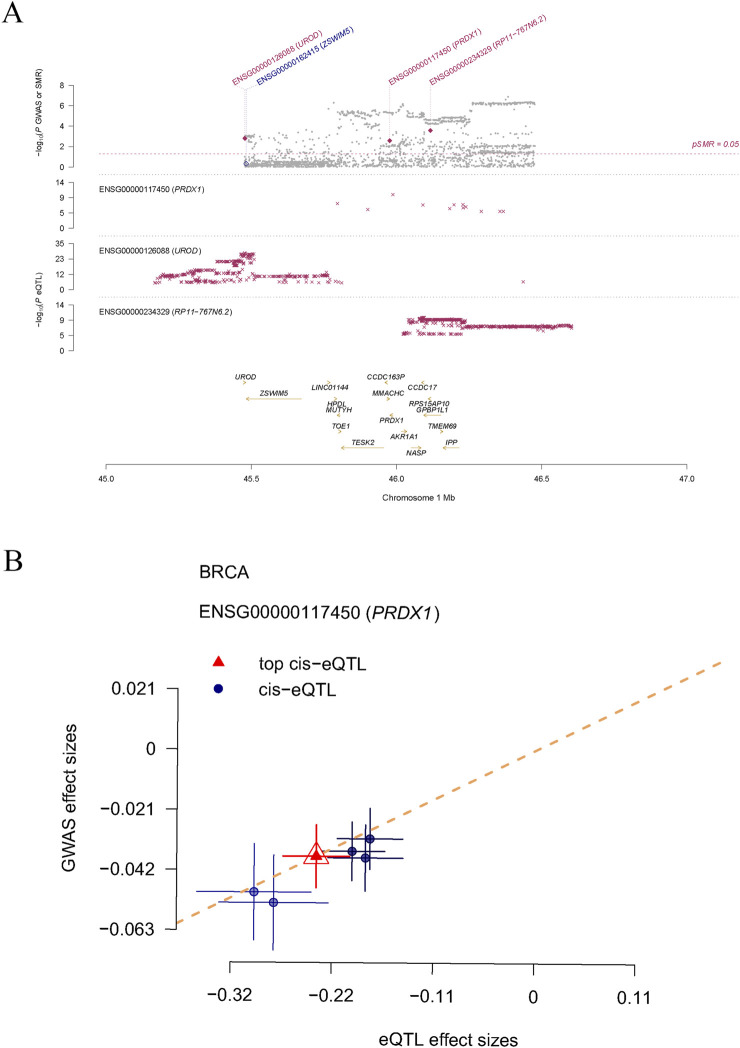
The causal relationship between LRGs and breast cancer, as determined by SMR analysis. **(A)** Displays three genes (PRDX1, UROD, and RP11-767N6.2) on chromosome 1 and their genetic association with eQTL. **(B)** Shows the positive correlation between the effect size of the PRDX1 gene’s eQTL and GWAS effect size.

In this study, we systematically identified a set of LRGs associated with BRCA, including DDX5, DHX16, LPPRC, MPHOSPH6, NSUN2, NUP50, PRDX1, RPL13, RPS23, and SRP14. Through rigorous statistical filtering, the PRDX1 gene exhibited significant results in SMR analysis (p_SMR = 0.002595542), while the HEIDI (Heterogeneity in Dependent Instrumental Variables) test revealed no considerable heterogeneity (p_HEIDI = 0.7630237). This important finding suggests that PRDX1 may play a critical role in the pathogenesis of BRCA. Therefore, we selected PRDX1 as the key gene for further analysis. Using the regional association plot generated by SMR analysis, we closely examined the genetic association of specific genes on chromosome one within the million base pair (Mb) region. The plot shows three genes on chromosome 1: ENSG00000117450 (PRDX1), ENSG00000126088 (UROD), and ENSG00000234329 (RP11-767N6.2), along with their association with expression quantitative trait loci (eQTL) (Figure 1A). In the correlation plot, the eQTL effect size of PRDX1 (i.e., the impact of genetic variation on gene expression) showed a positive correlation with the GWAS effect size (i.e., the impact of genetic variation on BRCA risk) (Figure 1B). Our findings suggest that PRDX1 may influence the development and progression of BRCA by regulating lactylation modification processes.

### 3.2 Expression and intercellular communication analysis of PRDX1 in BRCA single-cell RNA sequencing data

**FIGURE 2:**
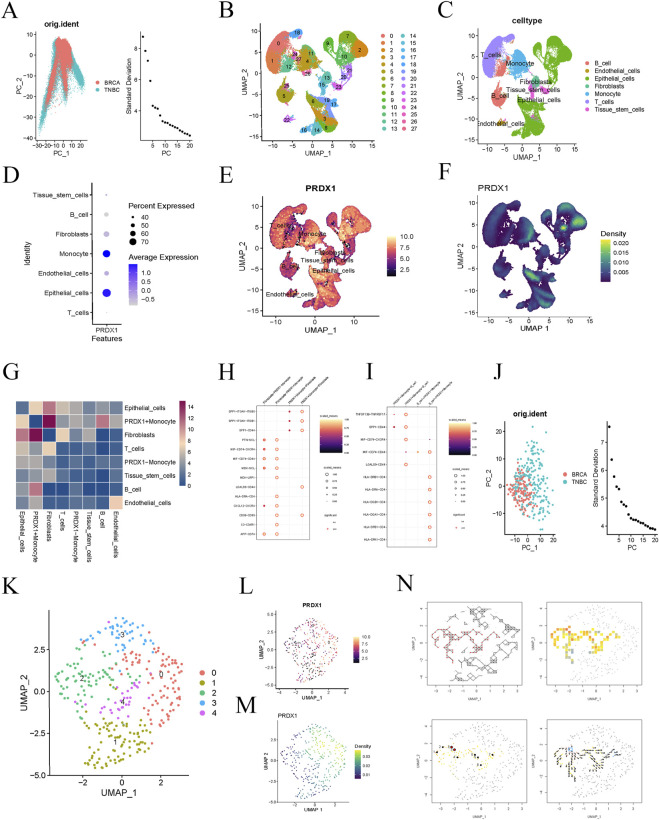
Single-cell analysis of BRCA patients. **(A)** PCA reveals differences in gene expression data distribution between BRCA and TNBC samples. **(B)** t-SNE clustering analysis identifies 27 distinct cell clusters. **(C)** Further analysis of the cell populations reveals seven cell types. **(D)** Bubble plot showing PRDX1 gene expression in different cell types. **(E, F)** Feature expression and density plots visualising the expression of the PRDX1 gene. **(G)** Cell-to-cell communication heatmap showing the communication activity between different cell types. **(H)** Cell communication network analysis indicates a potential specific communication pathway between PRDX1-positive monocytes and fibroblasts. **(I)** Cell communication network analysis indicates potential specific communication pathways between PRDX1-positive monocytes, fibroblasts, and B cells. **(J)** PCA plot of BRCA and TNBC samples after filtering for Monocyte cell subpopulations. **(K)** UMAP plot showing the distribution of cell clusters. **(L, M)** Visualisation of PRDX1 gene expression patterns in Monocyte cell subpopulations. **(N)** Pseudo-time analysis reveals the dynamic trajectory of Monocytes in the BRCA process.

We obtained a BRCA single-cell RNA sequencing dataset from the GEO database and selected gene expression data from 16 BRCA samples for in-depth analysis. We filtered the data during the initial quality control step and ultimately selected 3,000 highly variable genes for further analysis. PCA visualised the distribution of BRCA and triple-negative breast cancer (TNBC) samples (Figure 2A). The clustering analysis successfully identified 27 cell populations (Figure 2B). Upon further analysis of each cell population, we identified several distinct cell types, including B cells, endothelial cells, epithelial cells, fibroblasts, monocytes, T cells, and tissue stem cells (Figure 2C). Notably, the expression of PRDX1 was significantly increased in monocytes and epithelial cells, with an exceptionally high average expression level in monocytes (Figure 2D). Feature expression and density plots further visualised the same results (Figures 2E, F).

To further explore the function of PRDX1 and its role in intercellular communication, we classified monocytes into two groups based on PRDX1 expression: PRDX1-positive and PRDX1-negative. The intercellular communication significance heatmap analysis showed that PRDX1-positive monocytes exhibited significant communication activity with various cell types, suggesting their potential key role in the intercellular communication network. Furthermore, significant differences in communication patterns were observed between PRDX1-positive and PRDX1-negative monocytes, particularly in their interactions with fibroblasts and B cells. The heatmap indicated that the communication between PRDX1-positive monocytes and fibroblasts was the most significant (Figure 2G). By constructing an intercellular communication network, we further evaluated the communication probabilities between different cell populations, identifying key communication pairs and discovering potential specific communication pathways between PRDX1-positive monocytes and fibroblasts or B cells (Figures 2H, I). These pathways may be activated in specific physiological or pathological states, especially in tumor immune evasion, chronic inflammation, or immune diseases. Lactylated PRDX1-positive monocytes may promote the formation of an immune-suppressive environment through metabolic regulation, thereby enhancing their specific interactions with fibroblasts and B cells.

We then selected monocyte subpopulations and visualised the expression pattern of PRDX1 using UMAP dimensionality reduction and clustering analysis (Figures 2J–M). Using pseudotime analysis, we explored the dynamic trends of monocytes. Directional arrows visually represented the evolutionary trajectory of monocytes during disease progression (Figure 2N). This analysis not only enhanced our understanding of the role of monocytes in development, immune response, and disease progression but also opened up new research directions for exploring dynamic changes in cell fate determination and biological processes.

### 3.3 Spatial heterogeneity of PRDX1 gene expression and its intercellular communication patterns

**FIGURE 3:**
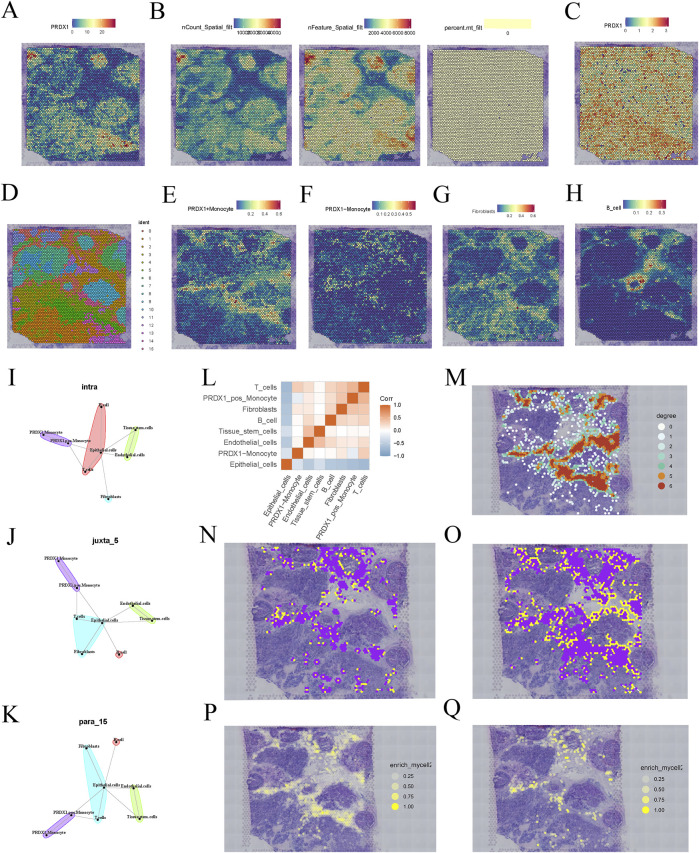
Spatial transcriptomics analysis of BRCA patients. **(A)** Expression pattern of PRDX1 gene in BRCA tissue sections. **(B)** Data quality filtering process for PRDX1 gene expression. **(C)** Spatial distribution of PRDX1 gene expression after normalisation and dimensionality reduction. **(D)** UMAP-based clustering results of cell populations. **(E–H)** Deconvolution analysis showing the distribution of PRDX1-positive monocytes, PRDX1-negative monocytes, fibroblasts, and B cells in tissue sections. **(I–K)** Communication relationships between different cell types under various communication modes (intra, juxta, para). **(L)** Heatmap of intercellular communication pairs, revealing significant communication pairs and network structure between cell populations. **(M)** Network diagram showing the homotypic cell network of PRDX1-positive monocytes in tissue sections. **(N, O)** Heterotypic cell network diagram between PRDX1-positive/negative monocytes and fibroblasts. **(P, Q)** Enrichment plot showing the interaction between PRDX1-positive/negative monocytes and fibroblasts.

The spatial gene expression distribution map shows the expression pattern of the PRDX1 gene in BRCA tissue sections (Figure 3A). The expression of PRDX1 exhibits significant spatial heterogeneity, with a higher concentration in the tumor nest region and relatively lower expression in the stromal region. This finding suggests that PRDX1 may play a more critical role in the BRCA microenvironment, with its expression closely associated with the biological behaviour of tumor cells. In contrast, its role in non-tumor regions may be more minor or distinct. To enhance the accuracy and reliability of subsequent analyses, we identified and excluded low-quality points (Figure 3B). After standardisation and dimensionality reduction, the spatial distribution map of PRDX1 gene expression was visualised (Figure 3C). Further clustering analysis revealed different cell populations and the distribution of cells within the tissue sections was visualised using UMAP (Figure 3D). We successfully integrated single-cell RNA sequencing data with spatial transcriptomic data by applying deconvolution analysis. Subsequently, we visualised the distribution of different cell types (PRDX1-positive monocytes, PRDX1-negative monocytes, fibroblasts, and B cells) within the tissue sections (Figures 3E–H). These visualisations clearly show that the distribution of fibroblasts closely matches that of PRDX1-positive monocytes in the tissue sections. This finding is consistent with previous single-cell transcriptomic analysis results. This integrated analysis method confirmed the consistency of intercellular communication patterns and highlighted the crucial value of spatial transcriptomic data in understanding complex intercellular interactions.

To further investigate intercellular communication, we applied MISTY analysis to quantify the intensity of communication between different cell types. The heatmap highlights significant communication pairs, showing stronger interactions between PRDX1-positive monocytes and the two cell types, fibroblasts and T cells (Figure 3L). Different spatial communication modes (intra, juxta, and para) also revealed that PRDX1-positive monocytes were near fibroblasts, T cells, and epithelial cells (Figures 3I–K). The homotypic cell network diagram revealed the distribution and spatial proximity relationships of PRDX1-positive monocytes within tissue sections, providing a clear view of intercellular interactions and tissue structure (Figure 3M). The heterotypic cell network diagram illustrated the spatial distribution of PRDX1-positive and negative monocytes and fibroblasts within the tissue (Figures 3N, O). The enrichment maps demonstrated the spatial enrichment of PRDX1-positive and negative monocytes and fibroblasts, with different colours representing varying degrees of enrichment (Figures 3P, Q). Through adjacency analysis, we found that PRDX1-positive monocytes tend to be adjacent to fibroblasts.

Previous studies have shown that fibroblasts, particularly cancer-associated fibroblasts (CAFs), can promote tumor invasion and immune evasion by remodelling the extracellular matrix (ECM) and secreting pro-tumorigenic factors (Zhang P. et al., 2025). Our findings indicate that PRDX1-positive monocytes tend to be located adjacent to fibroblasts, suggesting their potential role in tumor-stroma interactions. This close spatial association implies that PRDX1-positive monocytes and fibroblasts may shape the tumor microenvironment through reciprocal signalling, thereby influencing tumor progression.

### 3.4 Construction and validation of a BRCA survival prediction model based on PRDX1-positive monocyte gene expression

**FIGURE 4:**
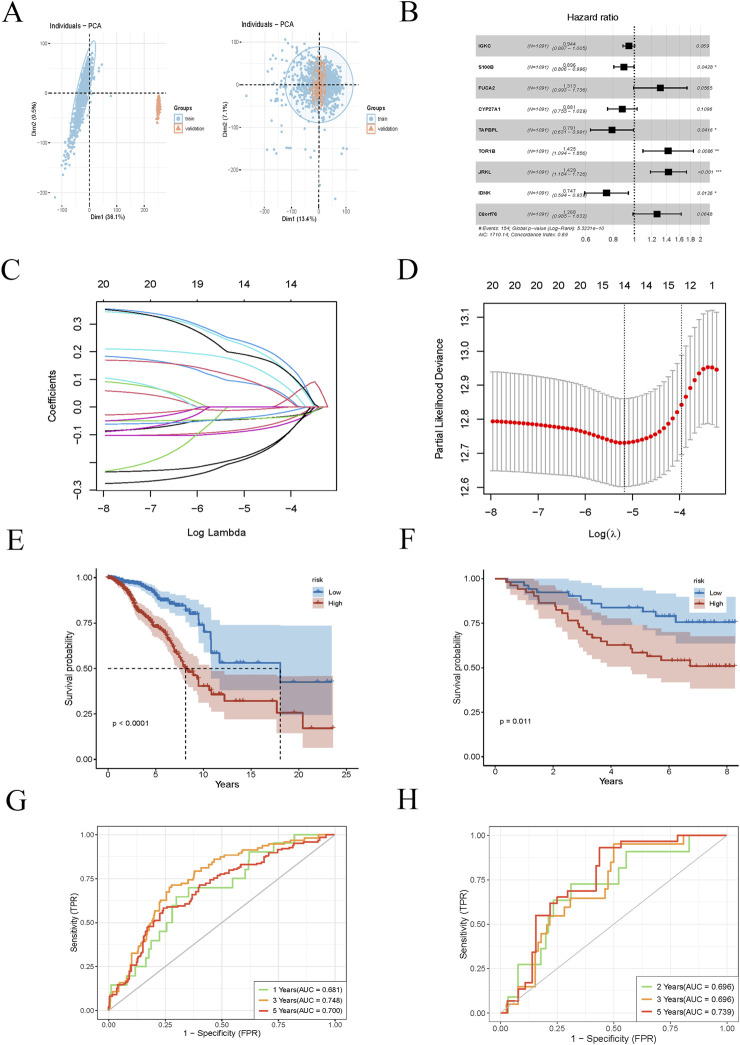
Prognostic model construction for BRCA patients. **(A)** Batch effect correction plot for BRCA samples from TCGA training set and GEO testing set. **(B)** Univariate Cox regression analysis identifies significant genes associated with BRCA survival. **(C, D)** LASSO regression model plot and cross-validation are used to select the optimal regularisation parameter (lambda). **(E, F)** Kaplan-Meier survival analysis curves for different risk groups of BRCA patients in the training and testing sets. **(G, H)** ROC curves for training and testing sets for 1-year, 3-year, and 5-year survival rates.

We selected genes upregulated in PRDX1-positive monocytes relative to other cell populations, requiring a log2 fold change (logFC) of at least 0.6 (i.e., a 1.5-fold change). First, we selected 1231 BRCA samples from the TCGA database as the training set to identify disease-related gene expression patterns and their association with survival. We then chose 121 independent BRCA samples from the GEO database as the test set to verify whether our findings were widely applicable and had predictive power. The first batch effect of the two datasets was corrected (Figure 4A). We then performed univariate Cox regression analysis to identify genes associated with BRCA survival that were significant. We used a LASSO regression model for gene selection, optimising the regularisation parameter (lambda) through cross-validation (Figures 4B–D). Next, we conducted a multivariate Cox regression analysis to further enhance the model’s predictive performance. By calculating risk scores for BRCA patients, we classified patients into high-risk and low-risk groups. Ultimately, we identified C8orf76, IDNK, JRKL, TOR1B, TAPBPL, CYP27A1, FUCA2, S100B, and IGKC as core genes for predicting the survival of BRCA patients. The expression levels of these genes have a significant impact on the survival risk of patients and may be key factors in determining the prognosis of BRCA. We evaluated the model’s predictive performance in training and test sets. Kaplan-Meier survival analysis revealed that high-risk BRCA patients had significantly shorter overall survival (OS) in both cohorts (Figures 4E, F). Receiver operating characteristic curve (ROC) curves showed that the area under the curve (AUC) values for 1-year, 3-year, and 5-year survival were as follows: training set (0.681, 0.748, 0.700) and test set (0.696, 0.696, 0.739), demonstrating good predictive performance (Figures 4G, H). These results suggest that our model has high accuracy in predicting the 1-year, 3-year, and 5-year survival of BRCA patients.

### 3.5 Validation of risk score based on model genes for predicting BRCA survival

**FIGURE 5:**
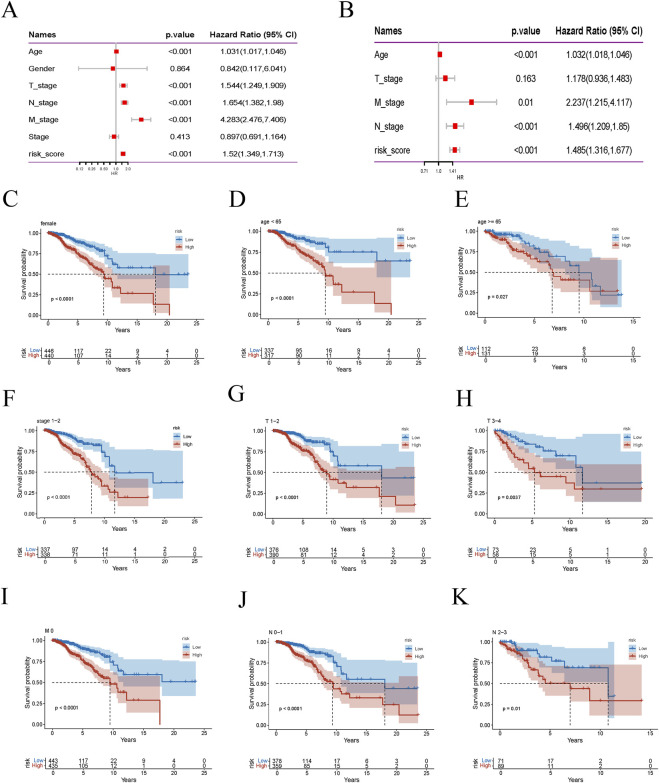
Risk score is highly correlated with clinical variables. **(A)** Forest plot of univariate Cox regression analysis. **(B)** Multivariate Cox regression analysis results. **(C–K)** Kaplan-Meier survival curves for different risk groups of BRCA patients stratified by age, gender, stage, T stage, M stage, and N stage.

We conducted a thorough univariate Cox regression analysis in the training set to identify significant associations between survival time and various clinical features, including age, T stage, M stage, N stage, and risk score. These associations were visualised using a forest plot (Figure 5A). The analysis revealed that age, T stage, M stage, N stage, and risk score were significantly correlated with survival time. Further multivariate Cox regression analysis confirmed that age, M stage, N stage, and risk score were independent predictors of survival time (Figure 5B). This indicates that the risk score, derived from the nine model genes, is a robust predictor of BRCA patient survival. Subsequently, we performed subgroup analyses stratified by different clinical features to evaluate survival differences within specific groups. Kaplan-Meier curves were used to compare the survival outcomes of low-risk and high-risk patient groups, stratified by age, gender, stage, and TNM classification (Figures 5C–K). The survival curve illustrates the groups where a significant survival difference was observed between the high-risk and low-risk groups (p < 0.05). These findings collectively validate the reliability and effectiveness of the risk score in predicting survival outcomes for patients with BRCA.

### 3.6 Immune suppressive microenvironment characteristics in high-risk groups

**FIGURE 6:**
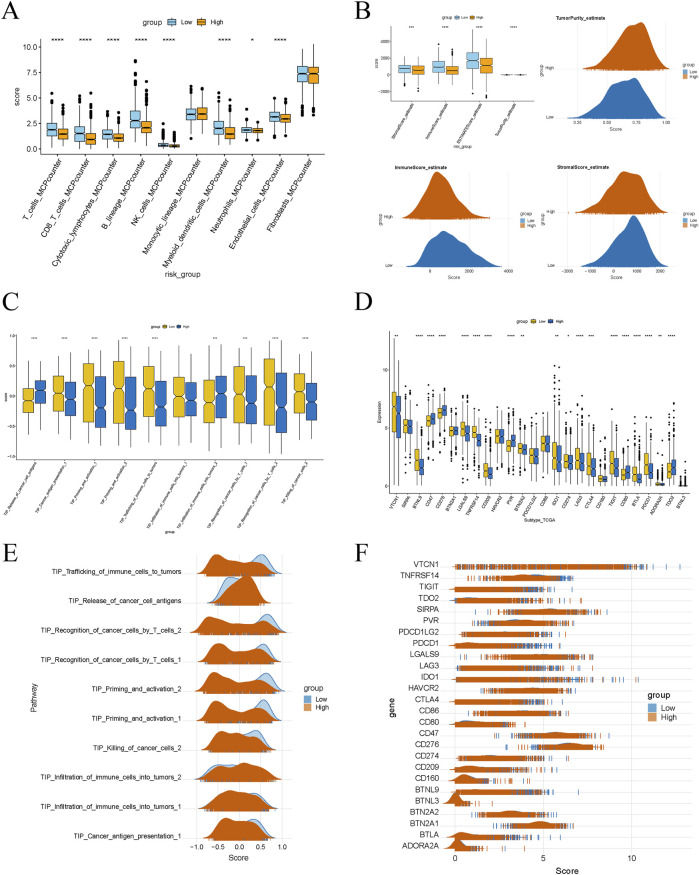
Significant differences in immune characteristics between different risk groups. **(A)** Box plot showing the difference in immune cell infiltration levels between high-risk and low-risk groups. **(B)** Box plot and peak plot showing significant differences in the stromal score, immune score, ESTIMATE score, and tumor purity between high-risk and low-risk groups. **(C, E)** Differences in immune-related pathway scores between high-risk and low-risk groups. **(D, F)** Differences in the expression of immune checkpoint genes between high-risk and low-risk groups.

Box plots revealed that the infiltration scores of T cells, CD8 T cells, cytotoxic lymphocytes, NK cells, myeloid dendritic cells, endothelial cells, and fibroblasts were significantly lower in the high-risk group compared to the low-risk group (Figure 6A), indicating an immune-suppressive phenomenon in the tumor microenvironment of the high-risk group. This suggests a marked inhibition of immune cell infiltration and function. The Matrix and immune scores, ESTIMATE composite scores, and tumor purity showed significant differences between the low- and high-risk groups (Figure 6B). Specifically, the high-risk group exhibited higher matrix scores, reflecting increased stromal cell infiltration. In contrast, lower immune and ESTIMATE composite scores indicated suppressed immune cell infiltration and function. Higher tumor purity in the high-risk group also suggested a more significant proportion of tumor cells and a relative decrease in stromal and immune cells. Scores for various steps in the Tumor-Infiltrating Immune Cells (TIICs) pathway were also lower in the high-risk group, including cancer cell antigen presentation, immune cell activation and migration, immune cell infiltration into the tumor, T cell recognition of cancer cells, and cancer cell killing (Figures 6C, E). This implies that key steps of the immune response in the tumor microenvironment are significantly inhibited in the high-risk group. Moreover, the expression levels of several immune checkpoint genes were significantly higher in the high-risk group, including VTCN1, SIRPA, BTNL9, CD47, CD276, and others (Figures 6D, F). The elevated expression of these genes may suppress immune cell activity, promoting tumor progression and potentially reflecting immune evasion mechanisms employed by tumor cells. These differences may serve as potential prognostic markers to predict disease progression and patient survival outcomes.

### 3.7 Experimental validation of PRDX1

**FIGURE 7:**
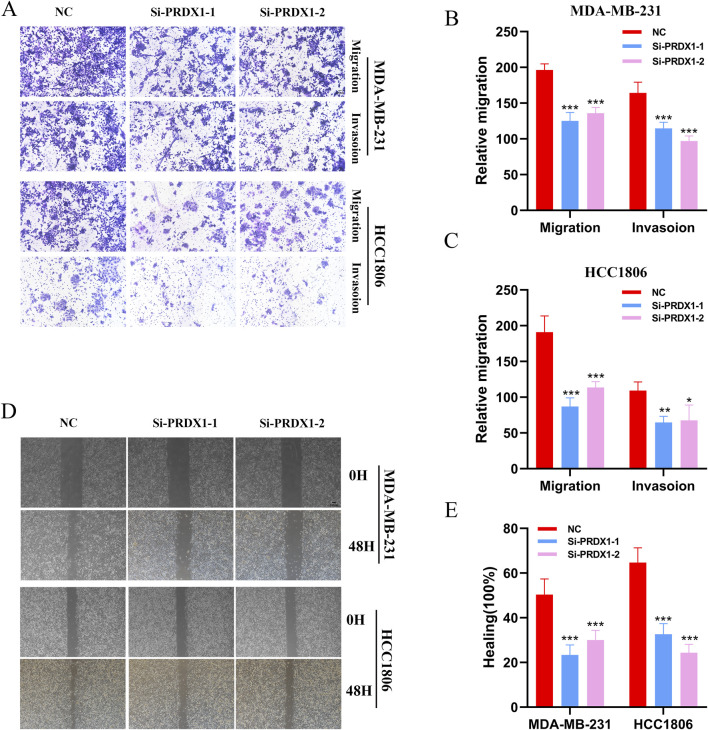
Function of PRDX1 in BRCA cells. **(A–C)** Transwell assays demonstrated the role of PRDX1 in cell migration and invasion. **(D, E)** Wound healing assays were performed at 0 and 24 h on HCC1806 and MDA-MB-231 cells to assess the role of PRDX1 in cell motility.

We conducted gene knockdown experiments in two cell lines. Using the Transwell assay, we found that MDA-MB-231 and HCC1806 cells with PRDX1 knockout exhibited markedly decreased proliferation rates when compared to their control counterparts (Figures 7A–C). Subsequently, we investigated the effect of PRDX1 on BRCA cell migration and invasion using wound healing assays. The findings revealed a substantial reduction in both migratory and invasive capabilities of BRCA cells following PRDX1 knockout (Figures 7D, E).

## 4 Discussion

This study comprehensively explores the regulatory mechanisms and prognostic value of LRG PRDX1 in BRCA through multi-omics analysis (Xie et al., 2023). Our findings highlight the significant role of PRDX1 in the initiation, progression, and involvement of BRCA in the immune microenvironment. Based on the gene expression profile of PRDX1-positive monocytes, we developed a survival prediction model, offering a new perspective for assessing BRCA prognosis.

We focused on the role of LRGs in BRCA, with a particular emphasis on PRDX1. Using the SMR method, we identified PRDX1 as a key LRG in BRCA. We discovered that it may play a crucial role in the genetic variation and regulation of gene expression related to BRCA risk. The expression of PRDX1 was significantly elevated in BRCA tissues, particularly in monocytes, indicating that PRDX1 may play a pivotal role in the BRCA-associated immune microenvironment (De Leo et al., 2024). PRDX1 is known to neutralise intracellular hydrogen peroxide, helping tumor cells survive under oxidative stress conditions. In the BRCA tumor microenvironment, PRDX1 is considered to play an essential role in immune cell regulation (He et al., 2025; Li et al., 2025). Further analysis revealed that the significant cell communication between PRDX1-positive monocytes and fibroblasts could potentially promote the immune evasion mechanism and immune suppression within the tumor microenvironment. Studies have shown that PRDX1 expression in monocytes, macrophages, and fibroblasts is closely related to the BRCA immune microenvironment, possibly regulating TAM function and contributing to immune evasion (Gao et al., 2022).

PRDX1’s role in various cancers is mainly associated with its antioxidant activity and immune regulation. In lung cancer (LC), PRDX1 protects cancer cells from damage by reducing oxidative stress and plays a significant role in chemotherapy resistance (Bai et al., 2023). Additionally, PRDX1 regulates the function of immune cells, promoting tumor immune evasion and influencing patient prognosis. In colorectal cancer (CRC), high PRDX1 expression correlates with tumor invasiveness, metastasis, and chemotherapy resistance, and its role in immune evasion and chemotherapy resistance makes it a potential therapeutic target (Song et al., 2024). In liver cancer (HCC), PRDX1 promotes cancer cell survival by reducing ROS accumulation and may exacerbate tumor progression by influencing immune evasion mechanisms (Hu et al., 2025). PRDX1 is also associated with tumor invasiveness, metastasis, and chemotherapy resistance in gastric cancer (GC) and other cancers such as pancreatic cancer (PC), ovarian cancer (OC), and prostate cancer (PCa), emphasising its essential biological function across various malignancies (Lai et al., 2024). Overall, PRDX1 plays a crucial role in tumor cell survival, metastasis, immune evasion, and chemotherapy resistance, making it a potential target for cancer treatment and immunotherapy.

Further spatial transcriptomic analysis revealed the spatial heterogeneity of PRDX1 gene expression, with high expression concentrated in the tumor nest region and lower expression in the stroma. This spatial distribution pattern may be closely linked to the biological behaviours of tumor cells, such as invasion and metastasis. Additionally, the spatial proximity between PRDX1-positive monocytes and fibroblasts suggests a close interaction between them (Wang et al., 2024). This interaction could influence the immune status of the tumor microenvironment and the remodelling of the extracellular matrix, thus promoting tumor progression.

Using the gene expression profile of PRDX1-positive monocytes, we constructed a survival prediction model for BRCA and validated it using TCGA and GEO datasets. The core genes of the model, such as C8orf76, IDNK, and JRKL, among others, demonstrated high accuracy in predicting survival outcomes for BRCA patients (Liu D. et al., 2025). This result confirms the importance of PRDX1-positive monocytes in BRCA prognosis and provides potential biomarkers for individualised prognostic assessment in clinical practice. Moreover, high-risk group patients displayed significant immune-suppressive microenvironment features, including reduced immune cell infiltration, high expression of immune checkpoint genes, and suppression of key immune response steps. These features suggest that PRDX1-positive monocytes may influence BRCA progression and prognosis by modulating the immune microenvironment.

However, several limitations remain in this study. First, the regulatory mechanisms of PRDX1 have not been fully elucidated, and its specific function in BRCA requires further validation through *in vitro* and *in vivo* experiments. Second, although the survival prediction model demonstrated good predictive performance in both the training and testing datasets, its applicability across different ethnic groups and clinical contexts requires further evaluation. Additionally, the spatial transcriptomic analysis was based on a limited sample size; future studies should expand the sample size to enhance the reliability of the results.

## 5 Conclusion

Through multi-omics analysis, this study reveals the regulatory mechanisms and prognostic value of the LRG PRDX1 in BRCA. PRDX1-positive monocytes exhibit significant intercellular communication activity and immune regulatory functions in the tumor microenvironment, and their gene expression profile can be used to construct an effective survival prediction model for BRCA. These findings provide a new theoretical basis and potential application directions for assessing BRCA prognosis and developing therapeutic targets.

## Data Availability

The datasets presented in this study can be found in online repositories. The names of the repository/repositories and accession number(s) can be found in the article/Supplementary Material.
