# Supplementary material for: Integrated multi-omics analysis reveals the functional and prognostic significance of lactylation-related gene PRDX1 in breast cancer
Source: Front Mol Biosci. 2025 Apr 4;12:1580622. doi: 10.3389/fmolb.2025.1580622 (PMC12006012; doi:10.3389/fmolb.2025.1580622)
Supplement: Supplementary file 1 [file Table1.docx]

| **Oligonucleotides** | **Nucleotide sequence (5'-3')** |
| --- | --- |
| **siRNA** |  |
| Scramble control | GCUUCGCGCCGUAGUCUUA |
| Si-PRDX1-1 | GATGAGACTTTGAGACTAGTT |
| Si-PRDX1-2 | GCTTTCAGTGATAGGGCAGAA |
|  |  |
| **Primer** |  |
| GAPDH | GGCCTCCAAGGAGTAAGACC (forward) |
|  | AGGGGAGATTCAGTGTGGTG (reverse) |
| PRDX1 | TCTCCAAGCAGAAGTGAGCG (forward) |
|  | GAAAGGCTGGTCTCTCCACC (reverse) |
|  |  |

**Table S1. Oligonucleotides used in research**
